# Neutrino intergalactic communication, metal life, and viruses: Part 1 quo vadis ex machina

**DOI:** 10.6026/97320630017331

**Published:** 2021-02-28

**Authors:** Paul Shapshak

**Affiliations:** 1 Division of Infectious Diseases and International Health, Department of Internal Medicine, University of South Florida, Morsani College of Medicine, Tampa, FL 33606, USA

**Keywords:** Machine life, exoplanets, Goldilocks, Astrobiology, metals, non-metals, ab initio, virus, origin, comets, asteroids, planetoids, moons, galaxies, stars, planets, panspermia, artificial intelligence, space exploration, electromagnetic radiation, neutrino, intergalactic and interstellar communication, psychology, paradigm, conflict

## Abstract

At one spectrum extreme, Astrobiology conjectures that for exoplanets with Goldilocks conditions, terrestrial-like life is inevitable. Moreover, it is envisaged that via panspermia, terrestrial-like life and its precursors are transferred among galaxies, stars, and within solar systems via transiting comets, asteroids, and planetoids. In addition, expelled stars, which have solar systems, it is inferred, transfer life as well. However, at the other extreme, we propose a paradigm shift that on some planets, subject to non- Goldilocks conditions, metal machine life could arise, ab initio, and evolve viruses, intelligence, and civilizations, conjointly. Accordingly, intelligent mechanized civilizations could readily and efficiently commence space exploration. Furthermore, as a counter paradigm shift, such civilizations could experiment and produce non-metallic life, based on carbon and other non-metal elements, under suitable conditions, related to Goldilocks life. Even a single example of validated interstellar or intergalactic communication received on the Earth would support the existence of life elsewhere. However, the communication platform should not be restricted to electromagnetic radiation. Other platforms should be included as well - one such example, which would require sophisticated technology, is neutrino communication. This is the case for any advanced civilization, be it metal-machine based, biological-based, and carbon-based. In sum, civilizations based on machine life, would be highly productive due to the longevity and hardiness of machine life. However, significant caveats are raised in this brief report, because possibly dissimilar psychologies and intelligence may lead to conflicts between metal machine life and biological life, inter-paradigm conflict.

## Background

A health crisis exists – this is nothing new in human history. For several thousand years, knowledge was lacking about disease, safety, and optimal health. Rampant infectious diseases led the list of virulent adversities, viruses included. However, viruses, though potentially noxious, are nevertheless a fundamental component of life. Nurturing our understanding of life and viruses is crucial for improving health, living standards, and treatment against disease. [1-4] In the 20th century, hypotheses and speculations were advanced as to how terrestrial life commenced, ab initio. This included a biochemical soup of spontaneously produced carbonaceous compounds, which included principally non-metal elements such as N, O, S, P, H, as well as C and a variety of metal-salts, water, coacervates, and clays. This work is exemplified in an extensive literature with numerous hypotheses and supportive experiments. [5-7] Consequent to these efforts, progress into the 21st Century resulted in the generalization of such ideas and findings - that terrestrial-like life on Goldilocks exoplanets abounds. Consequently, Goldilocks exoplanet exploration has commenced. In addition, there was a resurgence of the 19th Century promotion of panspermia. [8-14] There are approximately 100 billon stars in the Milky Way galaxy. However, an inventory of actual exoplanets, as of 12-25-2020, accumulated and tabulated by the US National Aeronautics and Space Administration (NASA), includes detection of 3,209 stars with planetary systems in the Milky Way; these contain 4,324 confirmed exoplanets and 5,695 candidate exoplanets. Moreover, 1,357 exoplanets are gas giants, 1,467 are Neptune-like, 1,331 are Super Earths; only 163 exoplanets are terrestrial (Goldilocks zone), and six more have as yet unspecified conditions. So, is there any life anywhere among these exoplanets and is life to be sought only among the Goldilocks planets? [8,9] Probably, exoplanet Goldilocks environments are favorable as habitats for terrestrial life; however, do Goldilocks habitats inevitably lead to terrestrial- like life, ab initio? A few decades into the 21st Century finds many scientists increasingly dissatisfied with Astrobiology notions that biological life inevitably originates, ab initio, only under Goldilocks conditions. Currently, we do not know how life originated on the Earth itself, neither have we detected life nor intelligence elsewhere. Furthermore, ascribing the 'first cause' to Panspermia is an oft-repeated corollary emblematic mechanism. Panspermia, it is speculated, may encompass spread of carbonaceous materials, ices, aquifers, as well as incipient or dormant life. However, it should not be ignored that a plethora of types of penetrating radiation (e.g., stellar prominences and winds, high energy particles, Xrays, UV) cause molecular degradation and destroy life. These results counter the Panspermia hypothesis. [1,9-17]

## Discussion:

Here, then, we propose an opposing hypothesis as to the origin of life on some exoplanets, ab initio, and its potential spread. A basic paradigm shift, we hypothesize that machine or mechanical life could arise, ab initio, on non-Goldilocks worlds, during the 13-billion-year existence of the universe. Several consequences follow. Intelligence, civilizations, and technology would then develop. Space exploration would proceed on their part, as well as interstellar communication. Machines would unmistakably have long life spans, rapid feasible repairs, and immense timescales to accomplish their tasks and goals.

A counter paradigm shift is that machines could utilize multitudinous methods to produce life under various conditions, including terrestrial-like Goldilocks conditions. In addition, and not unlikely, intelligent machine civilizations could further experiment, create, and promote Goldilocks life and its evolution. Moreover, machines could originate life on planets by placing basic primitive organisms (precursor-like life, stem cells) that have evolutionary potential. They could accomplish these experiments under various conditions of planets, including Goldilocks conditions, as well as environments that 20th Century Astrobiology considers extreme and adverse. On any such planet, life may develop, evolve, thrive, or become extinct when planets undergo changes in geology and climate. In addition, they are subject to changes in their star's evolution, and impacts from external sources, over time scales of millions of years. The ability of molecular metal machine life to replicate is at the base of the hypothesis. This is supported by theory and experiments since von Neuman's early 20th Century work in regard to self-replicating machines. In concert, increasing complexity and creativity of machines over time are not unexpected. [18,19]

Next, the question is how many elements - metal elements - are available for machine life compared to Goldilocks life. There are 118 elements; 94 (80%) of them are various classes of metals and 24 (20%) of them are non-metals. Thus, there are four-fold more metal elements compared to non-metals. Elements 91-118 are unstable. Two elements, Technetium (Tc, 43 protons) and Promethium (Pm, 61 protons) are absent from the Earth because all their isotope half-lives are shorter than the Earth’s age. However, the chemistry of metals is currently less understood than non-metals. Nonetheless, the number and types of bonding electrons among metals far exceeds those among non-metals. Thus, a much larger number of types of metal-compounds is anticipated than has been found for non-metals. Furthermore, organometallic molecules greatly increase the varieties of compounds available, which have intermediate properties. [17,20-22]

As we edge towards exoplanet exploration, our horizons should be open to broach new ideas, as to how life arose, ab initio. On planet Earth, in the first place, the origin of life remains a perplexing challenge. Furthermore, it is also unclear the extent to which Darwinian evolution may operate, away from planet Earth, as there could be other as yet undiscovered complexities that impact life-forms, be it metal or biological. Molecular transformation and changes in living forms might not be limited to processes of natural selection in the manner presented by Darwinists [23].

An example illustrating the difficulties imposed prior to and during the origin of life, is to ask, what are the probabilities of producing functional proteins from a mix or soup containing various ingredients, including amino acids? There is an average of 19.2 atoms per amino acid, and approximately 2,880 atoms in a chain of 150 amino acids. The probability is 10-57 to randomly and spontaneously produces a polypeptide chain that has a specific sequence of 150 amino acids. A process such as this to produce a specific sequence has a very low probability and low entropy. This implies an earlier series of multifaceted molecular mechanisms that must arise, prior to production of complex macromolecules and organelles. This problem includes proteins, enzymes, RNA, DNA, mitochondria, and chloroplasts, to name several. Darwinian selection probably operates to differing degrees at various levels, from molecular dynamics to genotype, phenotype, and biological dynamics. Similarly, it would be no less remarkable, by as yet additional unknown mechanisms, as to how metal molecule-based life may arise and evolve. The usual concepts of natural selection may not apply. [23]

Additional integral components of terrestrial life include viruses, which are obligatory parasites that infect terrestrial organisms, including eukaryotes, prokaryotes, and eubacteria. Nevertheless, viruses over-all are beneficial to many hosts. For example, many viruses frequently transfer host-genes among their hosts and viral genes themselves have ranges of effects on cellular molecular function. By analogy, then, metal-life could have the equivalent of viruses, small machines or nanomachines, which travel among diverse metal life forms, enhance their functions, perform repairs, modifications, enhance survival, and meet their environmental and societal challenges. [24]

Wherever life may exist, our possible communication with other civilizations in the Milky Way or in other galaxies would obviously support the existence of life beyond the Earth. Accordingly, what communication mechanisms are currently feasible and optimal for this purpose? Numerous forms of electromagnetic radiation of various wavelengths are used. However, in 1979, Pasachoff and colleagues, as well as others, proposed and have studied the use of neutrino detection for this purpose. Indeed, the use of neutrinos for interstellar and intergalactic communication is well founded because neutrinos are long-lived and highly penetrating, unlike electromagnetic radiation that is occluded by matter, dust-clouds, and many planetary and stellar atmospheres. (However, there are regions of some degree of transparency for electromagnetic radiation that are utilized for this purpose.) For neutrinos, of central importance, is their very small cross-section that also makes them difficult to detect; however, their detection technology continues to make great strides. Technology is also being improved to detect and resolve distant sources of neutrinos and measure their specific sub-types, energies, and masses.[25-32]

Neutrino analysis and characterization are widespread in contemporary physics and cosmology. Neutrino sources include stars, supernovae, and fundamental particle dynamics including radioactivity. Thus, additional complications in neutrino detection and their use for communication include their distribution among what are termed 'generations' or 'flavors' - neutrinos can transform (oscillate) from one form to another, over time, while traversing diverse distances. There are three flavors of neutrinos, termed electron, muon, and tau neutrinos. The fundamental equation that describes neutrino oscillation is:

 |n_a_>= Σj Uaj |n_j_> 

In this equation, neutrino flavors (electron neutrino ne, muon neutrino nm, and tau neutrino nt) are represented by |na> and are mass eigenstates; Uaj is a unitary transformation operator leptonic mixing matrix; and neutrino mass eigen states are represented by |n_j_> (j = 1, 2, 3). An unanticipated outcome of neutrino flavor oscillations includes its impact on extraterrestrial intelligence detection. This is because machine or other types of intelligence would be capable of evolving towards such capabilities. This complex illustration therefore exemplifies the subtleties required in the presumption that humans and the artificial intelligence produced there from, are capable of extraterrestrial communication. There is a high degree of complexity in developing neutrino detection and production as a means of communication. It is anticipated that machine life civilizations likely will be able to solve such intricate obstacles, as well. [25-29]

Recent developments in particle physics research involve neutrino entanglement as well as coherence. The three flavors of neutrinos are possibly interconnected by entanglement. Mass as well as flavor entanglement modes have been studied. Consequently, the above equation has been extended to time evolution, |n_a_(t)>, of the flavor neutrino state. t is time; i is the square root of -1; and Ej is the energy associated with each mass eigenstate |n_j_>.

 |n_a_(t)> = σj Uaj e∧(-itE) |n_j_> 

There is extensive debate as to whether entanglement exists; however, entanglements have been accomplished in several laboratories. If neutrino entanglements occur on cosmic scales, then that phenomenon may be utilized for intergalactic communications. It must be noted that there are differing views as to whether these methods are possible. [29-32]

Advanced civilizations could ostensibly utilize such properties of these fundamental particles to transmit communications, if they were motivated or able to do so. Interestingly, it is generally pre-supposed that extra-terrestrial advanced civilizations are out there, broadcasting their existence far and wide across the universe and that the bottleneck difficulty of technology development needed to acquire means to receive their signaling is ours alone. However, the obverse also may be the case - a reverse paradigm shift – that extra-terrestrial civilizations may select to hide from detection or there may be pronounced difficulties to accomplish broadcast status. Thucydides suggests hiding for intelligent civilizations that do not wish to risk annihilation. [33]

## Conclusions:

Currently, terrestrial scientific research and development advance towards production of living self-replicating machines with enhanced artificial intelligence. By contrast, in this paper, the hypothesis is advanced that living machines could arise, ab initio, on non-Goldilocks planets and that intelligence and machine civilizations could consequently proceed to develop. Therefore, signs of life and intelligence should be examined for non-Goldilocks adverse extreme environments as well as Goldilocks environments. Life and intelligence may be machine-like as well as biology-based and carbon-based. Additionally, neutrino-based communications may supersede electromagnetic communications and greatly expand the boundaries of extra-terrestrial intelligence existence and detection potential. A caveat of concern is that conflicts may occur among and between machine and biological civilizations. Even if machine civilizations evolved to solve the problems of conflict, that does not preclude human directed conflictive approaches nor does that preclude the uses of artificial intelligence and human-made machines to continue its history of conflict. [14,30-36] One curious consequence is how will human machine-made artificial intelligence interact with natural machine intelligence. Significant differences would be anticipated between intelligence derived from machine life and artificial intelligence derived from human-created machines. The topics presented in this brief hypothesis-review are listed in Table 1 (See PDF) with several representative references, where possible.
